# Characterization of Indoor Extremely Low Frequency and Low Frequency Electromagnetic Fields in the INMA-Granada Cohort

**DOI:** 10.1371/journal.pone.0106666

**Published:** 2014-09-05

**Authors:** Irene Calvente, Cristina Dávila-Arias, Olga Ocón-Hernández, Rocío Pérez-Lobato, Rosa Ramos, Francisco Artacho-Cordón, Nicolás Olea, María Isabel Núñez, Mariana F. Fernández

**Affiliations:** 1 University of Granada, San Cecilio University Hospital, Instituto de Investigación Biosanitaria ibs.Granada, Granada, Spain; 2 Unit Research Support of the San Cecilio University Hospital, Instituto de Investigación Biosanitaria ibs.Granada, Granada, Spain; 3 Department of Radiology, University of Granada, Granada, Spain; 4 CIBER en Epidemiología y Salud Pública (CIBERESP), Madrid, Spain; Institute for Health & the Environment, United States of America

## Abstract

**Objective:**

To characterize the exposure to electric fields and magnetic fields of non-ionizing radiation in the electromagnetic spectrum (15 Hz to 100 kHz) in the dwellings of children from the Spanish Environment and Childhood-“INMA” population-based birth cohort.

**Methodology:**

The study sample was drawn from the INMA-Granada cohort. Out of 300 boys participating in the 9–10 year follow-up, 123 families agreed to the exposure assessment at home and completed a specific *ad hoc* questionnaire gathering information on sources of non-ionizing radiation electric and magnetic fields inside the homes and on patterns of use. Long-term indoor measurements were carried out in the living room and bedroom.

**Results:**

Survey data showed a low exposure in the children's homes according to reference levels of the International Commission on Non-Ionizing Radiation Protection but with large differences among homes in mean and maximum values. Daytime electrostatic and magnetic fields were below the quantification limit in 78.6% (92 dwellings) and 92.3% (108 dwellings) of houses, with an arithmetic mean value (± standard deviation) of 7.31±9.32 V/m and 162.30±91.16 nT, respectively. Mean magnetic field values were 1.6 lower during the night than the day. Nocturnal electrostatic values were not measured. Exposure levels were influenced by the area of residence (higher values in urban/semi-urban *versus* rural areas), type of dwelling, age of dwelling, floor of the dwelling, and season.

**Conclusion:**

Given the greater sensitivity to extremely low-frequency electromagnetic fields of children and following the precautionary principle, preventive measures are warranted to reduce their exposure.

## Introduction

Human exposure to electromagnetic fields from non-ionizing radiation (EMF-NIR) has increased over recent decades, raising concerns about possible adverse health effects, although these remain controversial [1]. Humans are immersed in an electromagnetic “bubble” due to the growing use of electricity-dependent technologies. Sources of residential exposure to EMF-NIR include high-voltage power lines, transformers, and domestic electrical installations that generally emit low-frequency (LF) or extremely low-frequency (ELF) radiation between 0 and 300 kHz. Individuals are also increasingly exposed to radio frequencies (RFs) from radio stations and mobile phone/WIFI systems, among others.

ELF electromagnetic fields were recently classified as possibly carcinogenic (2B group) by the International Agency for Research on Cancer (IARC), based on epidemiological studies of childhood leukemia [2]–[4]. Other types of adverse health effects of exposure are considered “not classifiable” because of insufficient or inconsistent information [5].

Exposure to electric and magnetic fields in the home is influenced by various factors, including the use of electrical appliances, amount of electrical current flowing through the earth in the electrical distribution board, power consumption in the neighborhood, and distance between dwellings and from the power distribution system, among others. The field strength is significantly reduced with greater distance from the source.

The walls and roofs of houses can reduce the exposure to electrical fields from external equipment (e.g., power lines) [6]–[8] but provide a lesser screening against magnetic fields.

The strength of magnetic field in a dwelling, which is determined by the use of energy by neighbors as well as by the occupants, varies according to the time of day and season of the year. Thus, magnetic fields are generally at maximum values between 6 pm and 8 pm and at minimal values during the night, and there are also seasonal variations [6]–[8].

All electrical equipment produces an electric field and a magnetic field when in use. Electrical energy in the home is low-voltage, generating an electric field of only a few volts per meter. However, it has been reported that long-term exposure to these levels in buildings that are well-equipped with wireless devices but have inadequate ventilation and inappropriate construction materials may be responsible for the so-called “sick building syndrome”, associated with semi-circular lipoatrophy and other conditions [9], [10]. Electric fields are strongest directly under high-voltage lines, where the conductors are closest to the ground [6]–[8]. Stronger magnetic fields are generated by some devices than by others. The decline in magnetic field strength with distance is much more pronounced in the case of common electrical devices than in the case of power lines [7]–[8]. The power distribution system is the main source of electromagnetic field exposure outside the home but contributes little to the electric field within due to the shielding effect of walls and roof, as noted above. Magnetic fields near power lines vary according to the season, the demand for energy, and the technical characteristics of the lines (e.g., the height of the pole). Even underground distribution lines produce electromagnetic fields, which pass through matter and are not diminished by soil, rocks, or concrete [6], [7].

Increasing concerns about the possibility of adverse effects of exposure have led to investigations designed to improve methods for measuring exposure to electromagnetic fields from non-ionizing radiation (EMF-NIR) in the ranges of extremely low and low frequency electric [(ELF-LF)-EF] and magnetic [(ELF-LF)-MF] fields, and several studies have characterized this exposure in recent years [6], [7], [11]–[16].

It has been documented that children may be especially susceptible to exposure to EMF-NIR [11], [17]; hence, there is a need to establish current levels of exposure in this age group [18].

The objective of this study was to characterize the exposure to electric and magnetic fields of NIR in the 15 Hz to 100 kHz frequency range in the homes of children from the Spanish Environment and Childhood-“INMA” birth cohort.

## Materials and Methods

### Study Population

The study sample was drawn from the INMA network, a population-based cohort study in different regions of Spain (Ribera d'Ebre, Menorca, Granada, Valencia, Sabadell, Asturias, and Gipuzkoa) that focuses on prenatal environmental exposures in relation to growth, development, and health from early fetal life through childhood. The INMA study protocol includes medical follow-ups of the children from birth through childhood as well as epidemiological questionnaires and biological sample collections [19].

**Figure 1:**
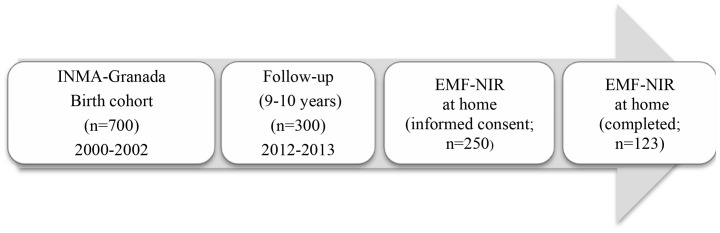
Chart depicting the flow of the children from recruitment at birth to final study subpopulation.

From October 2000 through July 2002, 700 eligible mother–son pairs registered at the San Cecilio University Hospital of Granada (province in Southern Spain) were recruited at delivery, establishing the INMA-Granada cohort. The inclusion and exclusion criteria were published elsewhere [20]. Between April 2005 and June 2006, one out of three families was randomly contacted to arrange a follow-up appointment, which included completion of an *ad hoc* questionnaire on their home environment [20]. Six years later (between January 2011 and December 2012), all families in the cohort (n = 700) were contacted and invited to participate in this follow-up. A total of 300 boys were finally enrolled and their families again completed *ad hoc* questionnaires on their home environment, including a specific questionnaire to gather information on the sources of EF-NIR and MF-NIR inside the home and on the patterns of use of electrical-electronic devices at home. Two hundred-fifty families signed informed consent to the performance of EMF-NIR measurements at home. The present study only included the 123 families/dwellings for which these measurements were finally carried out (Figure 1). The schooling of the parents was classified as primary, secondary, or university. A low educational level was reported by 41.5% of fathers and 44.7% of mothers. Only 26% of the fathers and 24.4% of the mothers had completed university studies.

### Ethical statement

We obtained written informed consent from the parents (mother or father) on behalf of children enrolled in your study. The 300 families registered in the follow-up signed the informed consent form, which included completion of ad hoc questionnaires. Two hundred-fifty out of three hundred families signed an additional informed consent to the performance of EMF-NIR measurements at home, but at the moment of the appointment became due, 127 families reneged on their decision. The study followed the guidelines laid down in the Declaration of Helsinki and was approved by the Ethics Committee of San Cecilio University Hospital, Granada, Spain.

### Characteristics of the study area

The setting of the INMA-Granada study is the health district of the San Cecilio University Hospital, an area of 4000 km^2^ with a total population of 512,000 inhabitants, including part of the city of Granada (236,000 inhabitants) and 50 towns and villages. Three areas of residence are differentiated: a) urban areas, corresponding to the city of Granada and towns with more than 20,000 inhabitants in the surrounding metropolitan area, b) semi-urban areas, towns with 10,000–20,000 inhabitants in the surrounding metropolitan area, and c) rural areas: small villages with less than 10,000 inhabitants. In the present study sample, 9.8% of households were in rural areas, 45.5% in semi-urban areas, and 44.7% in urban areas.

General characteristics of the households were as follows: 15.5% of families lived in detached houses, 45.5% in semi-detached houses, and 39% in apartments. The median age of the buildings was 15 yrs (range, 1.5 yrs to 62 yrs). The mean and median time of families in their current dwelling was 11 years (range, 0.16 to 28.0 yrs). Two rooms of the house were selected for ELF-LF measurements: the living room and the child's bedroom. Half of bedrooms (54.2%) were on the 3rd floor, 44.4% on the 2^nd^, and the remainder on the 1st floor. The living room was on the 2nd floor in 83.3% of the dwellings.

### Sources of electric and magnetic fields

The main sources of exposure to ELF-LF radiation in the living rooms and bedrooms were televisions, computers, music/DVD devices, electric braziers/radiators, heaters, air conditioning units, and energy-saving light bulbs. The largest proportions of electric-electronic devices were televisions (34%) and computers (32%), which were most frequently in the living room. Some type of energy-saving system (e.g., cold cathode fluorescent lamps) was used in the living room by 24.4% of families and in both rooms by 43.1%.

### Exposure assessment

The EMF-NIR is composed of two separate components: electric and magnetic fields. ELF-LF fields are associated with all aspects of the production, transmission, consumption, and transformation of electricity [11]. The assessment of exposure to [(ELF-LF)-EF] is generally more difficult and less well developed in comparison to the assessment of exposure to [(ELF-LF)-MF], because electric fields are easily perturbed by any conducting object, including the human body. Moreover, because there is no clear relationship between electric and magnetic fields in the near-field, both need to be assessed separately to determine electromagnetic exposure at a given point [11].

#### Equipment

Measurements were carried out for indoor sources using a Taoma base unit (Tecnocervizi, Rome, Italy), a broadband device with electric field and magnetic field isotropic probes with measurement ranges from 10 V/m to 100 kV/m and from 100 nT to 10 mT in the 15 Hz to 100 kHz frequency range. Quantification limits for electric [(ELF-LF)-EF] and magnetic [(ELF-LF)-MF] fields were 10 V/m and 100 nT (for the sum of all frequencies), respectively. These quantification limits are well below the most cautious guideline levels and therefore adequate for the purpose of the study; although some medical associations consider these limits to be too high for certain health problems associated with “electrosmog” [21]. Each probe is equipped with a temperature and humidity sensor. In the present study, the mean temperature ranged from 18.80 to 27.52°C and the relative humidity from 19.93 to 42.57%. The probe can be used while attached to the basic unit or connected by optical fiber cable to an Interface Box (I-Box) for automatic and autonomous data acquisition.

#### Measurement procedure

Long-term [(ELF-LF)-EF] and [(ELF-LF)-MF] measurements were performed every 240 s in the living room and child's bedroom, the areas at home where the children spent most time. The measurement procedure began with an initial exploration of the area of interest in order to identify punctual sources and to minimize perturbations caused by the proximity of the operator to the probe. Broadband measurements were then taken of electric and magnetic fields. The I-BOX and probes were placed on a non-metallic surface (desk/table in the center of the living rooms, and at the bed-side table [top end of the bed] in bedrooms) at an average height of 79 cm above the floor (based on the children's height at head and chest level). All devices in the household remained in their usual state during recordings, and there were no changes in the habitual internal sources. In a pilot study of 10 homes, the exposure was characterized on three different days. However, because virtually no difference was observed among the measurements on the different days, it was decided to perform the measurements on one day alone in the main study. In order to characterize everyday life exposure to all sources, measurements were made over a total of 17 h/day (between 3 pm and 10 pm in the living room and between 10 pm and 8 am in the bedroom) during a typical working day between October and June during the two-year study period.

### Covariates

An *ad hoc* questionnaire was used that comprised the following three sections: 1) socio-demographic and socioeconomic characteristics of the family (children and parents): age, years of education, residential history and characteristics, parental occupation history, and household income; 2) sources of exposure to EMF: possession and usage of telephones, wireless devices, and household equipment (computer, TV, air conditioner, refrigerator, etc.); and 3) information on the duration (in hours) of the use of each appliance/device. Data on the area of residence (urban, semi-urban, rural), type of residence (detached house, semi-detached house, or apartment), characteristics of dwelling (date of completion of construction, floor number, duration of occupation, and some ELF generating sources/devices such as televisions, computers, energy-saving lamps, and electric heating systems) and season (date of measurement) were finally used as covariates in this work.

### Statistical analysis

Descriptive analysis of measurements was performed, computing arithmetic means and standard deviations (SDs), median values, 5% trimmed mean values (after omitting lowest and highest 5% of measurements), and 25^th^ and 75^th^ percentiles. Comparison between variables was performed using the non-parametric Kruskal-Wallis test (χ2) and the Mann-Whitney U test. P≤0.05 was considered significant.

All measurements were performed by a single operator (I.C.). Excel 2010 and SPSS version 18 (IBM, Chicago, IL) were used for the data analyses.

## Results

**Figure 2:**
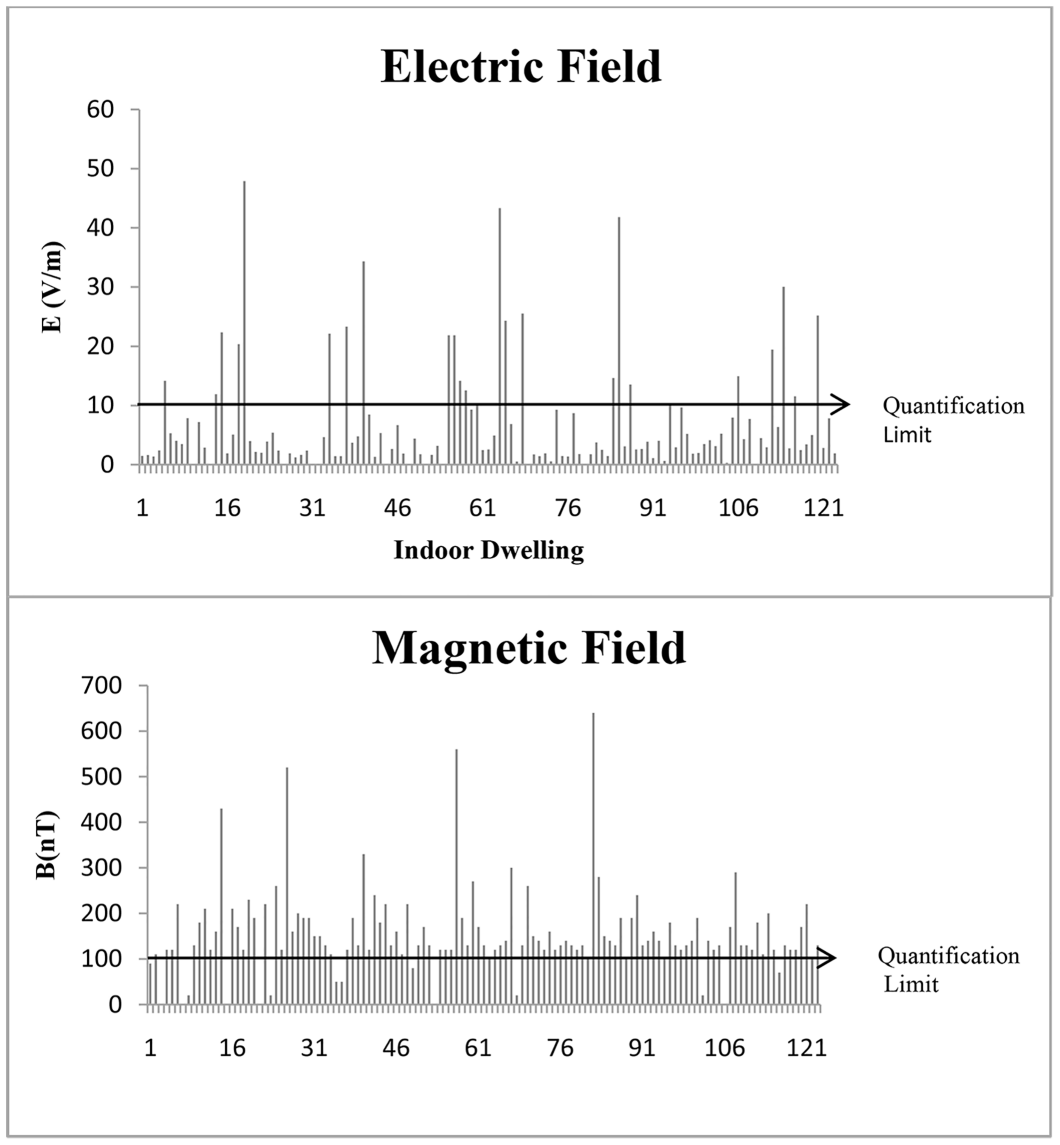
Distribution of A: extremely low frequency to low frequency electric field values, and B: magnetic field values.

Table 1 shows the levels of (ELF-LF)-EF and (ELF-LF)-MF exposure in the 123 participating families/dwellings. ELF-LF measurements had to be discarded in 6 out of the 123 dwellings due to recording faults, leaving a final study sample of 117 dwellings. The EF and MF exposure values found were very low, below ICNIRP guideline levels, while the (ELF-LF)-EF levels were highly variable in comparison to (ELF-LF)-MF values (Figure 2).

**Table 1:** Results of Measurements at home (n = 117).

Field	AM±SD	GM±SDG	Median	5% TM	p25	p75
**Electric [(ELF-LF)-EF] (V/m)**						
Day 3pm–10pm	7.31±9.32	2.54±9.30	3.68	6.08	1.84	8.58
Maximum	16.74±20.51		9.17	13.97	5.15	21.03
Minimum	2.78±4.91		1.25	2.01	0.01	2.17
**Magnetic [(ELF-LF)- MF] (nT)**						
Day (3pm–10pm)	162.30±91.16	142.53±1719	134.20	152.70	120.00	188.3
Maximum	1177.39±2375.34		685.00	859.70	445.50	1245.0
Minimum	42.23±22.22		49.00	42.42	36.00	56.00
Night (10pm–8am)	103.00±30.66		99.70	100.50	91.80	108.30
Maximum	476.00±2278.57		149.00	169.3	141.50	162.00
Minimum	44.25±21.03		43.00	44.30	33.00	57.50
Day-night (3pm–8am)	128.20±43.70		116.40	124.60	105.30	140.60
Maximum	1217.35±2280.36		788.00	907.67	461.50	1365.0
Minimum	37.83±21.51		42.00	37.54	26.50	52.50

nT: nanoTeslas; V/m: Volts/meter; AM: Arithmetical Mean; SD: Standard Deviation; GM: Geometrical Mean; SDG: Standard Deviation Geometrical; TM: Trimmed mean; p: percentile.

### Day-time measurements (3 pm to 10 pm)

#### (ELF- LF) Electric field

ELF-LF exposure levels were below the quantification limit of the probe (10 V/m) in 92 dwellings (78.6%). The arithmetic mean ±SD (ELF-LF)-EF value in the 117 dwellings was 7.31±9.32 V/m (above this mean value in 29.06% of dwellings), and the geometric mean was 2.54±9.30 V/m. The mean maximum value was 16.74±20.51 V/m and mean minimum value was 2.78±4.91 V/m; 25% of measurements were below 1.84 V/m or above 8.58 V/m (Table 1). In the 25 dwellings showing values within the measurement range of the probe, the arithmetic mean value was 22.05±10.52 V/m; 44% of measurements were above this mean, and the maximum value was 47.91 V/m (data not shown). Figure 2A depicts the distribution of ELF- LF values.

#### (ELF-LF)-Magnetic field

The arithmetic mean ±SD (ELF-LF)-MF value for the 117 dwellings was 162.30±91.16 nT (above this mean value in 38.46% of dwellings) and the geometric mean value was 142.53±1719 nT. The mean maximum value was 1177.39±2375.34 nT and the mean minimum value was 42.23±22.22 nT; 25% of the measurements were below 120 nT or above 188.30 nT (Table 1). Values were above the quantification limit of the probe (100 nT) in 108 dwellings (92.31%), which showed an arithmetic mean of 171.9±87.89 nT (data not shown). Figure 2B depicts the distribution of measurements among the 117 dwellings studied.

### Nocturnal measurements (10 pm to 8 am)

#### (ELF-LF) Magnetic field

**Figure 3:**
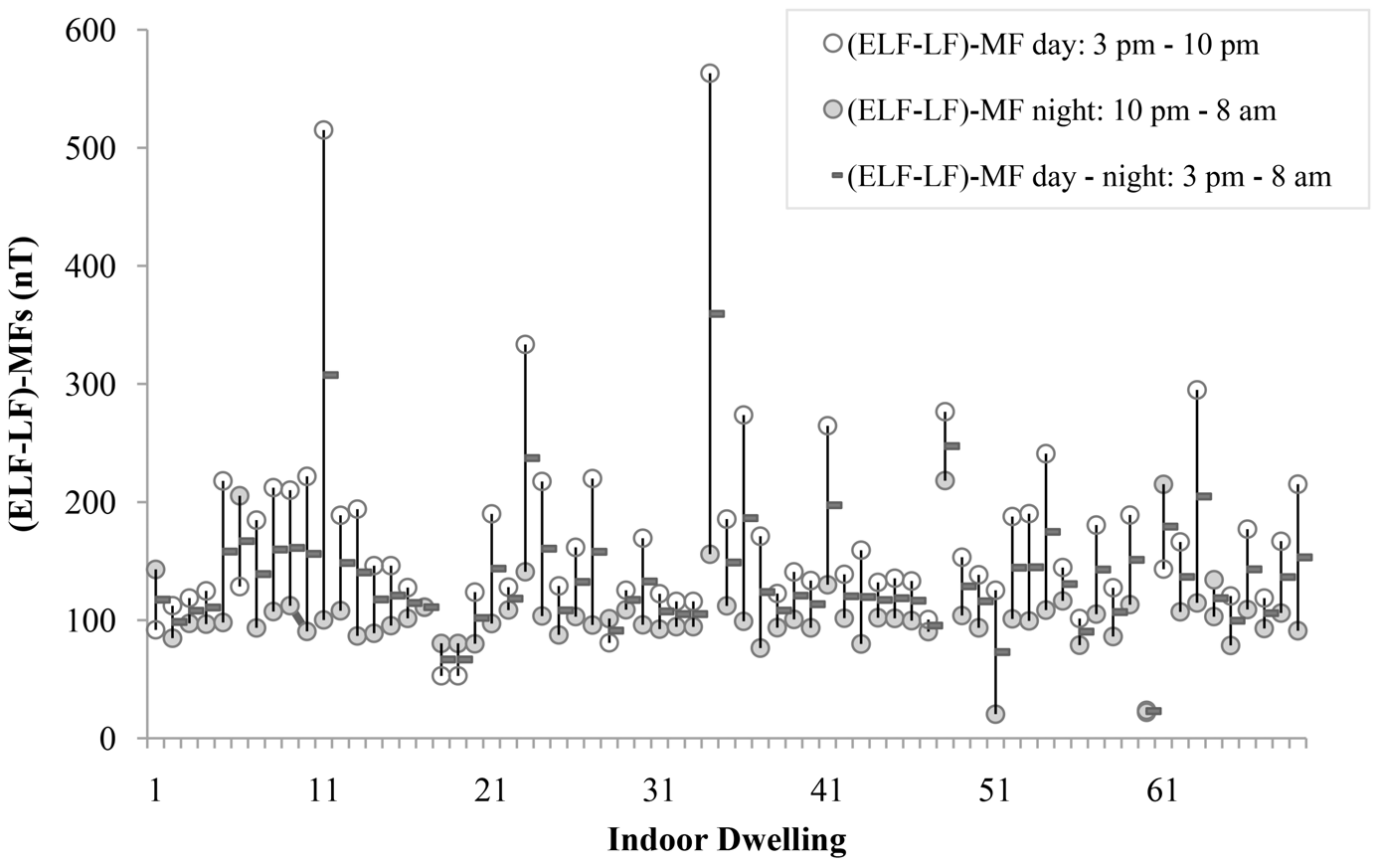
Daytime and nocturnal levels of exposure to extremely low frequency to low frequency magnetic fields [(ELF-LF)-MF].

Nocturnal measurements were only performed for magnetic field values. The arithmetic mean was 103.00±30.66 nT, i.e., 1.6-fold lower than daytime values, with 92% of measurements above the mean value and a geometric mean of 92.11±26.02 nT. The median value was 134.20 nT in the daytime and 99.70 nT at night. Median minimum values were similar between daytime and nocturnal measurements (Table 1). The arithmetic mean for the total exposure period (day plus night) was 128.20±43.70 nT, with 75% of measurements being above 105.30 nT and below 140.60 nT (Table 1). Figure 3 depicts the relationship of exposure to (ELF-LF)-MF between daytime and nocturnal measurements; these data were only available for 69 dwellings due to difficulties in maintaining the battery charge for the necessary time period (3 pm to 8 am). It can be observed that the variability in measurements was greater during the day than at night, when values appeared to be stable.

### Determinants of ELF-LF exposure

Table 2 describes the relationships of (ELF-LF)-EF and (ELF-LF)-MF exposure values with selected covariates. Daytime exposure to (ELF-LF)-EF and nocturnal exposure to (ELF-LF)-MF were significantly higher in urban and semi-urban *versus* rural areas and in apartments *versus* detached or semi-detached houses. The mean daytime (ELF-LF)-EF value was 2.01-fold higher (p = 0.024) and the mean nocturnal (ELF-LF)-MF value was 1.02-fold higher (p = 0.027) in urban and semi-urban *versus* rural settings.

**Table 2:** Summary of exposure to extreme low frequency-low frequency levels by area, type of dwelling, and indoor sources.

	EF (V/m) Day: 3 pm–10 pm						MF (nT) Day: 3 pm–10 pm						MF (nT) Night: 10 pm–8 am					
	n	mean	sd	p25	p75	p	n	mean	sd	p25	p75	p	n	mean	sd	p25	p75	p
**Area of residence**						**0.024**						**0.100**						**0.027**
Rural	12	3.83	6.24	1.43	2.88		12	136.00	65.13	105.00	178.10		10	100.90	42.23	80.30	98.65	
Urban/semi-urban	105	7.71	9.55	1.89	9.27		104	165.60	93.83	121.90	188.70		59	103.30	28.72	93.60	108.60	
**Dwelling**						**0.005**						0.189						**0.007**
Semi-detached/Attached	71	6.26	9.26	1.62	5.38		70	156.80	89.97	116.70	186.40		36	99.80	325.6	86.30	102.70	
Apartments	46	8.95	9.27	2.77	13.68		46	171.30	94.10	125.10	189.60		33	106.50	28.53	96.40	116.00	
**Age of the home**						0.072						**0.025**						0.841
<15 yrs	48	6.48	9.71	1.65	7.37		49	172.60	85.00	123.90	190.10		28	106.90	31.60	93.50	108.20	
≥15 yrs	55	8.20	9.50	2.13	10.11		53	145.60	82.54	115.50	160.40		32	104.00	32.94	88.20	108.60	
**Time living**						0.732						0.876				0.00		0.930
<9 yrs	36	7.25	10.60	1.84	7.37		35	171.80	108.72	121.80	190.00		24	104.20	26.53	93.50	107.40	
≥9 yrs	81	7.34	8.90	1.80	8.97		81	158.60	83.42	118.30	188.30		45	102.30	32.92	89.60	108.30	
**Floor number**						0.081						0.843						**0.041**
<3^a^	92	6.58	8.71	1.77	7.78		91	159.40	80.29	118.70	190.00		50	100.80	28.35	89.90	107.10	
≥ 3^a^	25	10.01	11.06	2.25	14.16		25	174.20	125.54	123.60	184.10		19	108.80	36.24	99.70	112.30	
**Sources-Devices**																		
**Television time use**												0.487						
≤1 h	26	6.15	9.31	1.71	8.66		26	175.00	61.10	126.2	222.9							
>1 h	90	7.59	9.38	1.89	8.13		90	158.4	98.55	118.0	188.0							
**Computer time use**						0.774						0.802						
≤1 h	105	7.14	9.39	1.81	7.89		105	164.50	93.83	120.20	189.10							
>1 h	11	8.52	9.23	1.86	19.42		10	136.7	59.53	101.60	185.8							
**Electric braziers**						0.532						0.395						
No	78	7.48	9.48	2.28	7.73		78	163.10	86.66	118.20	119.10							
Yes	39	6.99	9.10	1.65	10.11		39	160.7	100.72	121.10	157.30							
**Radiators**						0.967						0.608						0.348
No	65	6.80	8.31	1.81	8.58		64	155.10	71.69	121.30	175.10		61	103.70	31.81	93.50	108.3	
Yes	51	8.08	10.57	1.88	11.57		52	170.00	111.16	118.70	193.10		7	98.70	22.03	84.7	113.2	
**Heat-pump/air con**						0.409						0.394						0.089
No	46	7.40	8.80	1.98	10.53		44	144.10	57.32	119.10	183.40		54	102.40	34.12	89.90	107.50	
Yes	71	7.26	9.70	1.75	7.84		73	173.30	105.35	120.00	191.50		15	105.10	12.39	9.97	112.30	
**Energy-saving lamps**						0.666						**0.011**						0.634
No	40	6.36	6.78	2.43	7.83		35	138.00	79.64	115.70	156.50		28	100.90	28.99	90.80	107.90	
Yes	77	7.81	10.40	1.75	9.88		82	172.70	94.19	122.5	194.80		41	104.40	32.03	92.30	108.70	
**Season**						0.852						**0.036**						0.099
Spring-Summer	66	6.98	8.73	1.98	7.87		65	150.60	79.57	116.60	174.10		42	101.90	37.36	86,20	108.60	
Autumn-Winter	51	7.74	10.10	1.75	9.25		51	177.80	103.59	125.30	201.00		27	104.70	15.88	100,60	108.10	

Air-con: Air-conditioning; mean: arithmetical mean; SD: standard deviation; p25: Percentile 25; p75; Percentile 75; MF: Magnetic field or magnetic induction; EF: electric field; nT: nanoTeslas; V/m: Volts/meter.

Daytime (ELF-LF)-MF values were significantly higher (p = 0.025) in younger *versus* older dwellings, whereas daytime (ELF-LF)-EF values were significantly higher in older *versus* younger dwellings. Nocturnal (ELF-LF)-MF values were significantly higher (p = 0.041) in rooms on the 3rd floor or above (mean ±SD: 108.80±36.24 nT) *versus* rooms on lower floors (100.80±28.35 nT). No significant differences were found as a function of the time for which the family had occupied the dwelling.

Daytime (ELF-LF)-MF values were significantly higher in the spring/summer than in the autumn/winter (p = 0.036). A similar but non-significant tendency was observed for daytime (ELF-LF)-EF and nocturnal (ELF-LF)-MF exposure levels.

## Discussion

In this study, we characterized the exposure of children to EFs and MFs of NIR by performing long-term (daytime and nocturnal) measurements in the electromagnetic spectrum (15 Hz to 100 kHz) in the dwellings of children belonging to the INMA-Granada birth cohort. The EF and MF values found were very low, below International Commission on Non-Ionizing Radiation Protection (ICNIRP) guideline levels [22]–[23], and demonstrated a high variability.

As far as we know, the present study is the first to measured children's exposure to long-term (ELF-LF)-EF and (ELF-LF)-MF within their homes throughout lengthy daytime and nocturnal periods. Various approaches have been used to assess exposure to electromagnetic fields, including spot or long-term measurements, personal exposimetry/dosimeters, and the characterization of exposure based on activities and sources [6], [11]–[17]. These differences hamper the comparison of results among studies.

(ELF-LF)-EF levels were generally below the quantification limit of the probe (10 V/m). Overall exposure (ELF-LF)-EF values were lower than residential values reported in Austria [24], [25]. They were within the range of mean values reported in Europe [26] and similar to those recorded in a primary school in northern Spain [11]. However, they were higher than levels recently described by Huang et al. in two primary schools in Guangzhou, China [27].

(ELF-LF)-MF values were above 100 nT in 92.31% of daytime measurements, 63.77% of nocturnal measurements, and 86.96% of overall measurements. Mean residential ELF-MF levels have been reported to range between 25 nT and 70 nT in Europe and between 55 nT and 110 nT in the USA [26], [28]. The mean (ELF-LF)-MF values were higher than those described by Tomitsch et al. in Austria [24], [25] and by the WHO [26] but in the same order of magnitude as other reports [29]–[31]. Thus, Brix et al. used personal dosimeters to measure (ELF-LF)-MF exposure in subjects under 18-yr-olds and recorded mean values of 121±170 nT, with 25th and 75th percentiles of 41 nT and 143 nT, respectively, similar to the present findings [29]. Discrepancies among studies may be attributable to differences in sampling strategies and in the localization, height, or orientation of the probe, among other factors.

We distinguished between daytime (3 pm–10 pm) and nocturnal (10 pm–8 am) exposure measured in the living room and child's bedroom, finding that mean (ELF-LF)-MF values were 1.64-fold higher during the day (169 nT) than at night (103 nT). Various authors have reported higher day-time than nocturnal measurements [30]–[32], although one study [29] found lower exposure during the day (50 nT) than at night (92 nT), which was attributed by the authors to the influence of electric alarm clocks (58 nT without this device).

Mean ELF-LF measurements were higher in dwellings in urban or semi-urban *versus* rural settings, as reported by other authors [25], [29], [32], although this may be influenced by differences among the types of dwelling in the distinct areas, given that measurements were higher in apartments than in detached houses, also consistent with previous reports [25], [29].

Lower values were recorded in spring-summer than in autumn-winter, likely attributable to the greater use of electric heating and/or storage heaters during the colder months. Straume et al. also found differences in mean ELF-MF measurements between the summer (30 nT) and winter (70–80 nT) in public outdoor spaces in Norway [33].

Relationships between ELF-LF levels and specific sources (televisions, computers, heaters, etc.) were not consistent in our study, although a stronger association was observed when multiple domestic electrical and electronic devices were considered together (data not shown).

Study limitations include the relatively small sample size, the lack of data on individual exposure, and the fact that only 21.36% of electric measurements were within the range of the instrument. Moreover, the statistical power of the study was reduced by the application of non-parametric tests, although significance was reached (p<0.05). A study strength is that a single researcher was responsible for measuring levels in all dwellings and for gathering and analyzing all data. In addition, real measurements were analyzed, rather than estimates. The fact that the sample was drawn from an ongoing birth cohort also opens up the possibility of comparing exposure data with future health outcomes.

Various epidemiological studies have estimated that the risk of leukemia is two-fold higher in children who are exposed at home to ELF-MF levels above 300-400 nT [2]–[4], [34], [35]. Studies in 2001 and 2003 found that less than 1% of European children were exposed to residential exposure levels above 400 nT [34]. In the present study, however, daytime exposure reached >400 nT for 3.42% and >300 nT for 9.40% of the children, respectively; while nocturnal exposure was >300 nT for 2.40% of the children, although it never exceeded 400 nT. It should also be taken into account that the ICNIRP reference levels relate to short-term exposure, whereas the present results reflect long-term exposure. Given the greater sensitivity to ELF of children and following the precautionary principle [23], [36], preventive measures are warranted to reduce their exposure. One study found that the provision of specific recommendations to reduce field strengths in the home (e.g., unplugging devices not in use) led to a decrease in ELF-EF exposure levels [24].

## Conclusions

This study applied a detailed and accurate measurement protocol to characterize the indoor exposure of children to ELF-LF electric and magnetic fields at home. Residential exposure levels were below ICNIRP reference levels, but there was a wide variability in mean and maximum values, with 9.4% of the children receiving daytime exposure of >300 nT. There is a need for further studies of long-term exposure and for detailed research on its relationship with health outcomes.

## Supporting Information

File S1 **Informed consent.**
(DOC)
