# Supplementary material for: Characterization of Indoor Extremely Low Frequency and Low Frequency Electromagnetic Fields in the INMA-Granada Cohort
Source: PLoS One. 2014 Sep 5;9(9):e106666. doi: 10.1371/journal.pone.0106666 (PMC4156360; doi:10.1371/journal.pone.0106666)
Supplement: File S1 — Informed consent. (DOC) [file pone.0106666.s001.doc]

**Estudio INMA: Infancia y Medio Ambiente**

**Investigador principal: Nicolás Olea**

**Hospital Universitario San Cecilio**

**Aceptación voluntaria para participar en el estudio de la medida de radiación no ionizante en el interior de su vivienda**

(Copia Padres)

Cómo usted ya conoce, el proyecto de investigación que lleva por título “INMA (Infancia y Medio Ambiente): Exposiciones pre y postnatales a contaminantes ambientales, dieta, crecimiento fetal y desarrollo neuro-inmuno-endocrinológico” se realiza para estudiar el impacto de los contaminantes ambientales sobre la salud de los niños.

Para poder ampliar el conocimiento sobre los efectos de la contaminación sobre la salud, es necesario conocer, el papel de la radiación no ionizante en la predisposición a ciertas enfermedades.

Si usted acepta participar en esta parte del proyecto, se realizarían próximamente medidas de radiación no ionizante en el interior de su vivienda. El procedimiento a seguir es el siguiente: Se coloca en la habitación donde el niño pasa más tiempo y sobre una superficie firme no metálica, dos aparatos autónomos que miden la radiación no ionizante, con dos sondas respectivamente y permanece en ese lugar de 24h como máximo. Este aparato no necesita de ningún tipo de conexión a red eléctrica y es inocuo. Tendremos que pasar por su casa a colocar los aparatos y a recogerlos finalizada la medición.

Toda la información relacionada con este estudio será anónima de forma que será imposible relacionarla con su hijo/a. Solo investigadores relacionados con el estudio tendrán acceso a los datos, que serán tratados de forma confidencial según la legislación vigente (Ley Orgánica 15/1999, de 13 de diciembre, de protección de datos de carácter personal). Los resultados del estudio pueden ser publicados en revistas científicas.

Recordatorio: Factura de la luz; diagrama de barras del consumo de un año por meses


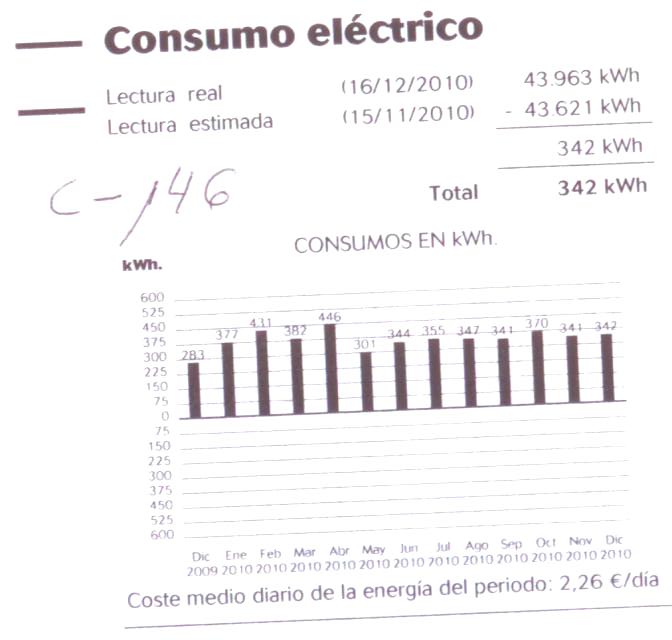


**Estudio INMA: Infancia y Medio Ambiente**

**Investigador principal: Nicolás Olea**

**Hospital Universitario San Cecilio**

**Aceptación voluntaria para participar en el estudio de la medida de radiación no ionizante en el interior de su vivienda**

(Copia Padres)

Yo, .................................................................................................. (nombre y apellidos),

- He leído la información que se me ha entregado.
- He podido hacer preguntas sobre esta parte del estudio.
- He recibido suficiente información sobre esta parte del estudio.
- He hablado con **el responsable**, quien me ha aclarado las dudas.
- He sido informado que los resultados de esta parte del estudio serán anonimizados de modo que será imposible relacionarlos con mi hijo/a.
- Comprendo que la participación de mi hijo/a es voluntaria.
- Comprendo que el estudio está diseñado para incrementar los conocimientos médicos.

Presto libremente mi conformidad para que se realicen medidas de radiación no ionizante en el interior de mi vivienda.

Nombre y apellidos............................................................................................

DNI: ..............................................................

Firma

Lugar y fecha: ............................................, a ____ de ........................ de 201__

Firma del responsable

Nombre y apellidos...............................................................

**Estudio INMA: Infancia y Medio Ambiente**

**Investigador principal: Nicolás Olea**

**Hospital Universitario San Cecilio**

**Aceptación voluntaria para participar en el estudio de la medida de radiación no ionizante en el interior de su vivienda**

(Copia INMA)

Cómo usted ya conoce, el proyecto de investigación que lleva por título “INMA (Infancia y Medio Ambiente): Exposiciones pre y postnatales a contaminantes ambientales, dieta, crecimiento fetal y desarrollo neuro-inmuno-endocrinológico” se realiza para estudiar el impacto de los contaminantes ambientales sobre la salud de los niños.

Para poder ampliar el conocimiento sobre los efectos de la contaminación sobre la salud, es necesario conocer, el papel de la radiación no ionizante en la predisposición a ciertas enfermedades.

Si usted acepta participar en esta parte del proyecto, se realizarían próximamente medidas de radiación no ionizante en el interior de su vivienda. El procedimiento a seguir es el siguiente: Se coloca en la habitación donde el niño pasa más tiempo y sobre una superficie firme no metálica, dos aparatos autónomos que miden la radiación no ionizante, con dos sondas respectivamente y permanece en ese lugar de 24h como máximo. Este aparato no necesita de ningún tipo de conexión a red eléctrica y es inocuo. Tendremos que pasar por su casa a colocar los aparatos y a recogerlos finalizada la medición.

Toda la información relacionada con este estudio será anónima de forma que será imposible relacionarla con su hijo/a. Solo investigadores relacionados con el estudio tendrán acceso a los datos, que serán tratados de forma confidencial según la legislación vigente (Ley Orgánica 15/1999, de 13 de diciembre, de protección de datos de carácter personal). Los resultados del estudio pueden ser publicados en revistas científicas.

**Estudio INMA: Infancia y Medio Ambiente**

**Investigador principal: Nicolás Olea**

**Hospital Universitario San Cecilio**

**Aceptación voluntaria para participar en el estudio de la medida de radiación no ionizante en el interior de su vivienda**

(Copia INMA)

Yo, .................................................................................................. (nombre y apellidos),

- He leído la información que se me ha entregado.
- He podido hacer preguntas sobre esta parte del estudio.
- He recibido suficiente información sobre esta parte del estudio.
- He hablado con **el responsable,** quien me ha aclarado las dudas.
- He sido informado que los resultados de esta parte del estudio serán anonimizados de modo que será imposible relacionarlos con mi hijo/a.
- Comprendo que la participación de mi hijo/a es voluntaria.
- Comprendo que el estudio está diseñado para incrementar los conocimientos médicos.

Presto libremente mi conformidad para que se realicen medidas de radiación no ionizante en el interior de mi vivienda.

Nombre y apellidos............................................................................................

DNI: ..............................................................

Firma

Lugar y fecha: ............................................, a ____ de ........................ de 201__

Firma del responsable

Nombre y apellidos...............................................................
